# SILVER FRIEND: A SMART SPEAKER PROJECT TO ASSIST LOW-INCOME OLDER ADULTS LIVING ALONE IN SOUTH KOREA

**DOI:** 10.1093/geroni/igz038.938

**Published:** 2019-11-08

**Authors:** Jongwoong Kim

**Affiliations:** University of Cincinnati, Cincinnati, Ohio, United States

## Abstract

Ownership rate of smart speakers in the U.S. reaches 21 percent in late 2018, and the growth of the global market is predicted to explode in the coming years. With this trend, there is an increasing expectation that smart speakers can assist older adults to stay independently in their homes as they age. However, there still is a shortage of empirical studies and evidence that these devices can actually be effective in helping older adults age in place. This paper describes a pilot project, Silver Friend, in South Korea, which utilizes a smart speaker, NUGU, to help low income older adults who live alone remain their homes as actively and safely as possible. The Silver Friend project has largely two goals: 1) reducing the older adults’ loneliness and 2) improving emergency response via the NUGU’s voice recognition and data monitoring system. Initially, the project served 90 older adults who live alone in government-purchased rental housing. This project was a result of a public-private partnership led by a South Korean tech/telecom giant, SK Corporation, practicing its corporate social responsibility. In South Korea, 15 percent of the population is age 65 and older, and 20 percent of them (approximately 1.5 million older adults) are living alone and/or in need of financial assistance. With the ever-expanding global market for smart speakers, this paper focusing on a case in South Korea describes evidence and a potential policy model in utilizing a smart speaker to help achieve the goal of aging in place.

